# AI detection of knee joint effusion from radiographs: Comparative accuracy of two commercial algorithms

**DOI:** 10.1016/j.ejro.2026.100760

**Published:** 2026-05-08

**Authors:** Jarno T. Huhtanen, Mikko Nyman, Roberto Blanco Sequeiros, Seppo K. Koskinen, Tomi K. Pudas, Sami Kajander, Pekka Niemi, Hannu J. Aronen, Jussi Hirvonen

**Affiliations:** a Faculty of Health and Well-being, Turku University of Applied Sciences, Finland; b Department of Radiology, University of Turku, Finland; c Department of Radiology, University of Turku and Turku University Hospital, Turku, Finland; d Terveystalo Inc, Jaakonkatu 3, Helsinki, Finland; e Department of Radiology, Tampere University, Faculty of Medicine and Health Technology and Tampere University Hospital, Tampere, Finland

**Keywords:** Artificial intelligence, Knee, Joint effusion, Radiograph

## Abstract

**Background:**

Knee joint effusion might indicate injury even without bony changes. Automated detection from radiographs could improve the sensitivity of AI algorithms.

**Purpose:**

To compare two commercially available AI algorithms, BoneView and RBfracture, in detecting knee joint effusion.

**Material and Methods:**

This retrospective study collected 123 lateral knee radiographs. Detection of knee joint effusion by both AI algorithms was compared with two board-certified radiologists with arbitration. Sensitivity, specificity, positive predictive value (PPV), negative predictive value (NPV), accuracy, and interobserver agreement (Cohen’s Kappa) were calculated. 95% confidence intervals (CI) assessed robustness. McNemar’s tests compared sensitivity and specificity between AI algorithms.

**Results:**

Knee joint effusion was present in 56% of radiographs. BoneView demonstrated a sensitivity of 0.42 (95% CI: 0.31–0.54), specificity of 1.00 (95% CI: 0.93–1.00), PPV of 1.00 (95% CI: 0.88–1.00), NPV of 0.57 (95% CI: 0.47–0.67), and accuracy of 0.68 (95% CI: 0.59–0.75). RBfracture demonstrated a sensitivity of 0.75 (95% CI: 0.64–0.84), specificity of 0.91 (95% CI: 0.80–0.96), PPV of 0.91 (95% CI: 0.81–0.96), NPV of 0.74 (95% CI: 0.63–0.83), and accuracy of 0.82 (95% CI: 0.74–0.88). Cohen’s Kappa was 0.49 (95% CI: 0.35–0.63), indicating moderate agreement between the two AI algorithms. Adding knee joint effusion detection to fracture/dislocation predictions improved sensitivity.

**Conclusions:**

Two commercially available AI algorithms demonstrated different operating points for knee joint effusion detection: BoneView achieved high specificity, while RBfracture achieved higher sensitivity. Combining injury and effusion predictions increased sensitivity at the cost of specificity.

## 1 Introduction

Knee trauma is common across all ages [1], and plain radiographs remain an important first-line imaging modality [2], [3]. Knee radiographs are among the most frequently performed radiographic examinations [4]. The knee joint is a complex anatomical region where effusion is a significant radiological finding, associated with intra-articular injury [5]. Knee joint effusion is seen as soft tissue density in the suprapatellar bursa in lateral radiographs [6] and can result from injury, degeneration, or inflammation [7]. Lipohemarthrosis, evident as the presence of fat and blood in the joint space, presents as a fat-fluid level in the pre-femoral region [8].

Detecting knee joint effusion can be challenging from radiographs [9]. Automated detection using artificial intelligence (AI) may help healthcare providers improve sensitivity and guide treatment [10]. Although AI has been widely used in detecting injuries in radiographs, few studies have focused on the detection of knee joint effusion in radiographs. Good sensitivity and specificity compared to human readers have been demonstrated [9], [11], but modest results have also been reported [10]. Overall, there is a tendency for AI to produce false-positive effusion predictions from radiographs [12] and moderate sensitivity and specificity values [13].

We have previously demonstrated high diagnostic accuracy and good agreement in injury detection from radiographs for two commercially available AI algorithms, BoneView (Gleamer) and RBfracture (Radiobotics) [14]. However, sensitivities in the knee were the lowest among all musculoskeletal (MSK) regions. Injury-associated knee joint effusion may be a clinically significant finding, indicating the possible presence of fractures or ligament tears [15]. We aimed to determine and compare the performance of these AI algorithms in detecting knee joint effusion. We included data from both children and adults, as well as multiple imaging devices, to address generalizability. In addition, we included both cross-table lateral projections, which have been shown to be highly sensitive for detecting knee joint effusion [16] and rolled lateral projections. To our knowledge, there is limited information on the comparison between different commercially available AI algorithms in interpreting knee joint effusion using the same dataset. We hypothesized that 1) the AI algorithms have high and comparable diagnostic accuracy in knee joint effusion detection, and 2) adding knee joint effusion predictions to fracture/dislocation predictions improves overall sensitivity for injury detection.

## 2 Material and methods

### 2.1 Study ethics

This retrospective study received ethical approval from the Ethics Committee of the University of Turku (ETMK Dnro: 38/1801/2020). The research complied with the Declaration of Helsinki, and due to its retrospective nature, the need for informed consent was waived by the Ethics Committee of the Hospital District of Southwest Finland.

### 2.2 Study population and design

The dataset included radiographs from our previous study collected between 2018 and 2021, all of which were annotated at the case level by two board‑certified radiologists specialized in musculoskeletal imaging with 20 and 25 years of experience. Their consensus assessment for fracture and dislocation were derived from our previously published study, while effusion ground truth (no effusion/effusion) was established independently using the same reference readers and a consensus approach. In effusion detection the reference readers only had patient age and radiographs without the knowledge of e.g. referral or possible other imaging modalities. In cases of disagreement, the opinion of a third radiologist was used. In this study, 'injury' refers to the radiographic finding of a fracture or dislocation. The AI algorithms were analyzed on the Collective Minds Radiology platform (Collective Minds Radiology, Stockholm, Sweden), a cloud-based system facilitating AI integration with radiographic data using diagnostic monitors. Radiographs from multiple imaging devices were included. Inclusion criteria were: (a) only patients referred for radiography because of a recent traumatic incident, (b) availability of an original radiology report from either a radiology specialist or resident, and (c) primary radiographs. Exclusion criteria included (a) projections not compatible with the AI algorithms, (b) lateral projections unavailable, and (c) follow-up studies. No examinations were incompatible with one AI but not the other.

### 2.3 AI algorithms used in this study

Two commercially available AI algorithms were evaluated: BoneView (Gleamer, Paris, France, v.2.5.1), RBfracture (Radiobotics ApS, Copenhagen, Denmark, v.2.2.1). Both BoneView and RBfracture are CE-marked for fracture, dislocation and effusion detection on radiographs. Cases were anonymized while retaining data compatible with AI software requirements, including patient age, and uploaded to the platform in DICOM format without preprocessing. Both AI algorithms independently analyzed the radiographs and generated binary outputs (no effusion/joint effusion present). In addition, any doubtful or low-confidence outputs from the AI algorithms were interpreted as abnormal. Data were processed in May 2025 to ensure compatibility with the platform’s latest software version. The AI algorithm outputs were recorded without knowledge of the ground truth or original reports.

### 2.4 Statistical analyses

AI algorithm predictions were compared against the ground truth used in this study to calculate and compare diagnostic accuracy metrics. Data were expressed in terms of true positives (TP), true negatives (TN), false positives (FP), false negatives (FN), and accuracy. Ninety-five percent confidence intervals (95%CIs) were determined using the Wilson score method. McNemar’s test examined differences for paired binary outcomes. Interobserver agreement between the AI algorithms was evaluated using Cohen’s unweighted Kappa, based on a 2 × 2 contingency table of their paired predictions. Kappa was calculated in R (version 4.4.0; R Foundation for Statistical Computing, Vienna, Austria) using the psych package, with 95%CIs provided to assess the precision of the agreement estimate. Categorical variables were summarized with counts and percentages, and associations were evaluated using chi-squared tests or Fisher’s exact test where appropriate. P values smaller than 0.05 (two-tailed) were considered statistically significant. To assess whether the inter-algorithm agreement reflects genuine case-level disagreement or a threshold offset, a logical-OR ensemble (positive if either algorithm flagged effusion) was compared with RBfracture alone using McNemar's exact test.

### 2.5 Exploratory analysis: combined injury and effusion prediction

As an exploratory analysis, we calculated the combined diagnostic accuracy for predicting knee joint effusion and injury by assigning the radiograph as abnormal if either was predicted by the AI algorithm and comparing this against the ground truth of injury findings.

## 3 Results

**Fig. 1:**
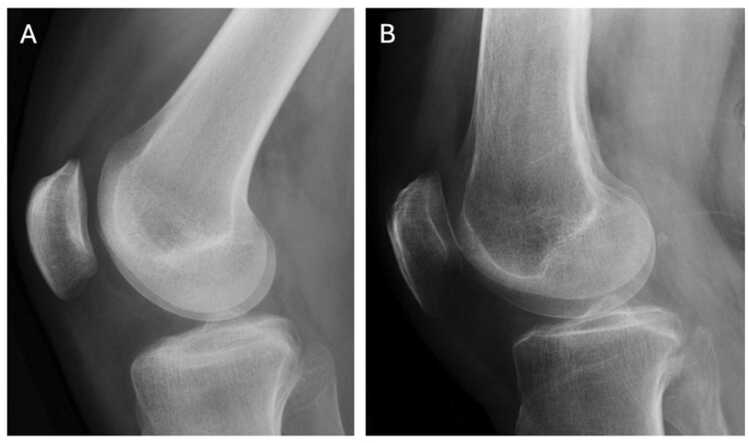
Examples of normal lateral knee radiograph with no joint effusion (A) and knee with joint effusion (B) based on the ground truth.

Among the 123 radiographs, 95 (77%) were cross-table lateral projections and 28 (23%) were rolled lateral projections. According to the ground truth, 69 radiographs (56%) had knee joint effusion, and 54 radiographs (44%) did not (Fig. 1). Regarding injury findings (fractures and dislocations), the ground truth identified 24 cases (20%) with injury and 99 cases (80%) without injury. As expected, knee joint effusion was observed more frequently in cases with injury findings (79%) than in those without (51%) (χ2=6.4, p = 0.011). There were 7 cases with disagreement in joint effusion evaluation and in these cases a third radiologist was used to solve the disagreement. Patient demographics are presented in Table 1.

**Table 1:** Patient demographics.

Demographic	All patients
Total	123
Pediatric (2–17yo)	26
Male	77
Female	46
Mean Age (SD)	41.5 (25.5)
Range	2–99
Injury prevalence	19.5% (24/123)
Joint effusion prevalence	56.1% (69/123)

*SD* standard deviation.

### 3.1 Diagnostic accuracy for knee joint effusion detection

**Fig. 2:**
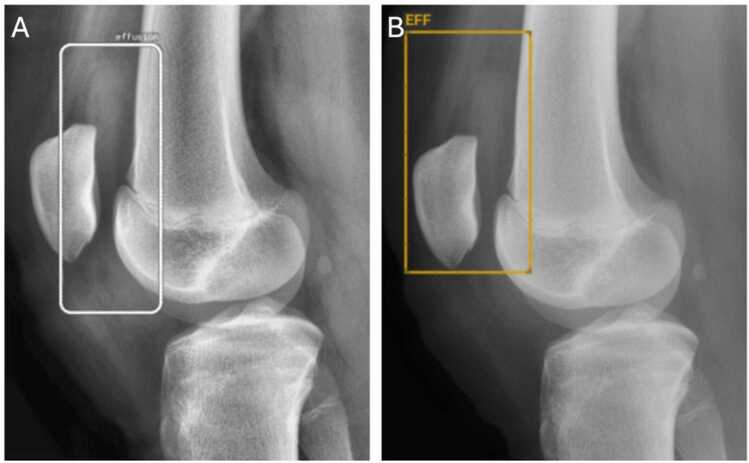
TP findings from RBfracture (A) and BoneView (B) in a patient with knee joint effusion based on the ground truth.

**Fig. 3:**
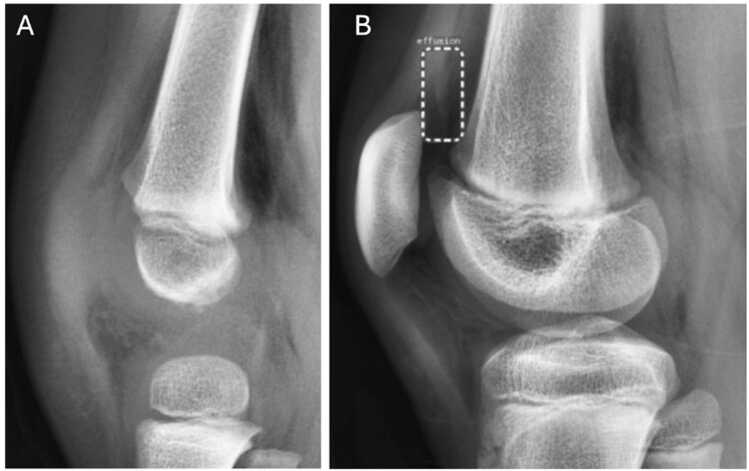
FN findings from both AI algorithms (A) in pediatric patient with knee joint effusion based on the ground truth. FP finding from RBfracture (B) in a patient with no knee joint effusion. BoneView demonstrated no FP findings in this study.

For knee joint effusion detection, BoneView demonstrated a sensitivity of 0.42 (95% CI: 0.31–0.54), specificity of 1.00 (95% CI: 0.93–1.00), PPV of 1.00 (95% CI: 0.88–1.00), NPV of 0.57 (95% CI: 0.47–0.67), and accuracy of 0.68 (95% CI: 0.59–0.75). RBfracture demonstrated a sensitivity of 0.75 (95% CI: 0.64–0.84), specificity of 0.91 (95% CI: 0.80–0.96), PPV of 0.91 (95% CI: 0.81–0.96), NPV of 0.74 (95% CI: 0.63–0.83), and accuracy of 0.82 (95% CI: 0.74–0.88) (Table 2). McNemar’s test indicated a significant difference in sensitivity between the two AI algorithms (χ2=19, p < 0.001), with RBfracture outperforming BoneView. However, the difference in specificity bordered on statistical significance (χ2 =3.2, p = 0.073). Examples of TP, FP, and FN for BoneView and RBfracture are shown in Fig. 2, Fig. 3.

**Table 2:** Algorithm performance metrics for knee joint effusion detection and combined injury and effusion detection.

	TP	TN	FP	FN	Sensitivity	Specificity	PPV	NPV	Accuracy	McNemar’s p (Sens)	McNemar’s p (Spec)	Cohen’s Kappa
Knee joint effusion detection												
BoneView (95% CI)	29	54	0	40	0.42 (0.31–0.54)	1.00 (0.93–1.00)	1.00 (0.88–1.00)	0.57 (0.47–0.67)	0.68 (0.59–0.75)	p < 0.001	p = 0.073	0.49 (0.35–0.63)
RBfracture (95% CI)	52	49	5	17	0.75 (0.64–0.84)	0.91 (0.80–0.96)	0.91 (0.81–0.96)	0.74 (0.63–0.83)	0.82 (0.74–0.88)			
Combined prediction (injury OR effusion)												
BoneView (95% CI)	22	79	20	2	0.92 (0.74–0.98)	0.80 (0.71–0.87)	0.52 (0.38–0.67)	0.98 (0.91–0.99)	0.82 (0.74–0.88)	p = 1.000	p < 0.001	0.61 (0.48–0.75)
RBfracture (95% CI)	22	57	42	2	0.92 (0.74–0.98)	0.58 (0.48–0.67)	0.34 (0.24–0.47)	0.97 (0.89–0.99)	0.64 (0.55–0.72)

### 3.2 Inter-algorithm agreement

Kappa for knee joint effusion detection between BoneView and RBfracture was 0.49 (95% CI: 0.35–0.63), indicating moderate agreement between the two AI algorithms. A logical-OR ensemble of BoneView and RBfracture achieved a sensitivity of 0.77 (95% CI: 0.66–0.85) and specificity of 0.91 (95% CI: 0.80–0.96). The ensemble added one true positive over RBfracture alone (53/69 vs 52/69; McNemar exact p = 1.00).

### 3.3 Impact of patient positioning

Stratification by lateral projection type (cross-table n = 95, rolled n = 28) revealed no significant influence of patient positioning on diagnostic performance (Supplementary Table S1). RBfracture consistently showed higher sensitivity than BoneView (full cohort: 0.75 vs. 0.42, p < 0.001; cross-table: 0.77 vs. 0.39, p < 0.001), while BoneView maintained perfect specificity across all subgroups. Confidence intervals overlapped substantially when comparing cross-table and rolled projections within each algorithm.

### 3.4 AI knee joint effusion detection and correlation to verified injury findings

**Fig. 4:**
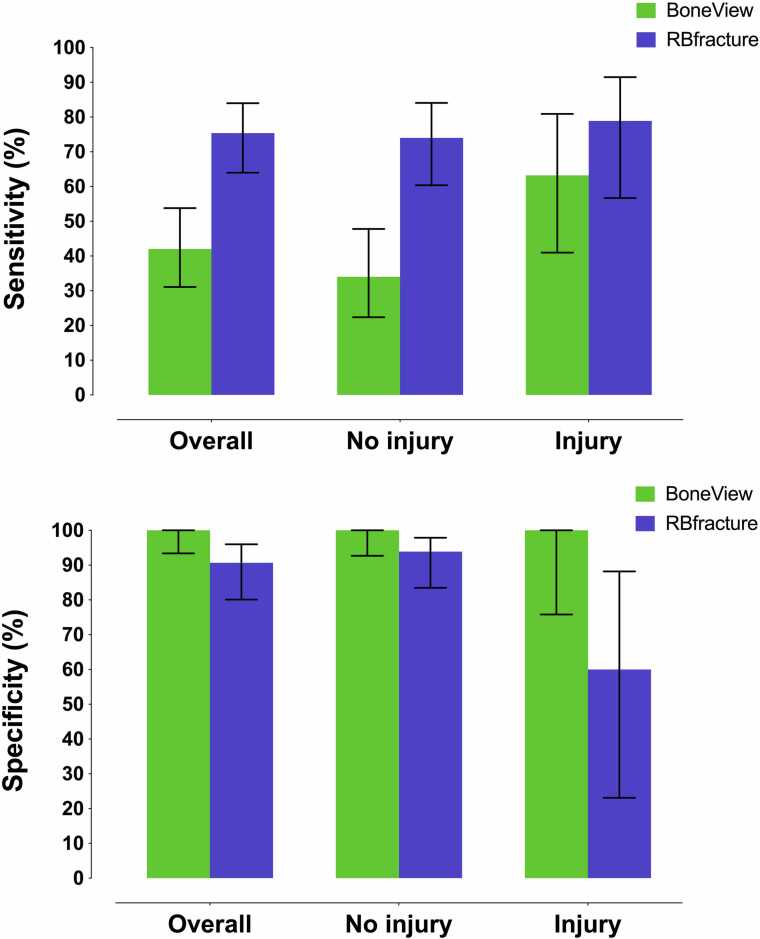
Sensitivity and specificity of AI algorithms in all cases and stratified into cases with and without injury findings (fractures or dislocations).

The performance of AI algorithms’ knee joint effusion detection varied based on the presence or absence of injury findings (fractures and dislocations) verified by ground truth. For BoneView, knee joint effusion was detected in 50% of cases with injury findings and in 17% of cases without (χ2 =9.8, p = 0.002). The values for RBfracture were 71% and 40% (χ2 =6.0, p = 0.014), respectively. In non-injury cases, BoneView exhibited lower sensitivity (0.34, 95% CI: 0.22–0.48) compared to RBfracture (0.74, 95% CI: 0.60–0.84) (p < 0.001) (Fig. 4). In injury cases, BoneView’s sensitivity increased to 0.63 (95% CI: 0.41–0.81), while RBfracture’s was 0.79 (95% CI: 0.57–0.92), with no significant difference (p = 0.371). BoneView maintained perfect specificity (1.00) in both classes, whereas RBfracture’s specificity dropped from 0.94 to 0.60 in injury cases. Finally, Cohen’s kappa for knee joint effusion detection between BoneView and RBfracture was 0.47 (95% CI: 0.31–0.63) in non-injury cases and 0.42 (95% CI: 0.08–0.75) in injury cases.

### 3.5 Combined injury and knee joint effusion predictions

**Fig. 5:**
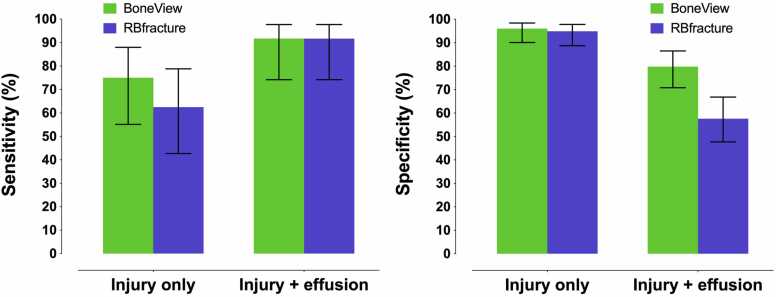
Sensitivity and specificity of AI algorithms in detecting overall injury using only injury findings (fractures or dislocations) and injury findings combined with effusion.

BoneView’s combined prediction achieved a sensitivity of 0.92 (95% CI: 0.74–0.98), specificity of 0.80 (95% CI: 0.71–0.87), PPV of 0.52 (95% CI: 0.38–0.67), NPV of 0.98 (95% CI: 0.91–0.99), and accuracy of 0.82 (95% CI: 0.74–0.88). RBfracture’s combined prediction also reached a sensitivity of 0.92 (95% CI: 0.74–0.98), with a specificity of 0.58 (95% CI: 0.48–0.67), PPV of 0.34 (95% CI: 0.24–0.47), NPV of 0.97 (95% CI: 0.89–0.99), and accuracy of 0.64 (95% CI: 0.55–0.72) (Fig. 5).

When the combined predictions were compared against predictions including only injury findings (fractures and dislocations), which were published in our previous work, sensitivity increased from 0.75 to 0.92 for BoneView and from 0.63 to 0.92 for RBfracture. McNemar’s test assessed whether the combined approach significantly improved sensitivity over injury-only predictions. For BoneView, the sensitivity increase was not statistically significant (χ2 =2.25, p = 0.134). For RBfracture, the improvement was significant (χ^2^=5.1, p = 0.023). Specificity and PPV decreased for both AI algorithms, with BoneView’s specificity decreasing from 0.96 to 0.80 and PPV from 0.82 to 0.52, and RBfracture’s specificity decreasing from 0.95 to 0.58 and PPV from 0.75 to 0.34, indicating increased false positives.

## 4 Discussion

The purpose of this study was to compare the diagnostic performance of two commercially available AI algorithms, BoneView and RBfracture, in detecting knee joint effusion from knee radiographs. BoneView demonstrated a sensitivity of 0.42, specificity of 1.00, and accuracy of 0.68, whereas RBfracture demonstrated a sensitivity of 0.75, specificity of 0.91, and accuracy of 0.82. The difference in sensitivity between AI algorithms reached statistical significance, whereas the difference in specificity did not, although specificity reached an inconclusive trend-level significance. We found moderate agreement between AI algorithms. Finally, combining knee joint effusion with injury predictions increased sensitivity for both algorithms, at the cost of specificity and positive predictive value. These exploratory findings should be interpreted in the context of the limited sample size and require prospective validation.

Our study showed more modest sensitivity values for BoneView compared to previous studies using non-commercial algorithms [9], [10], [11] in knee joint effusion detection, while RBfracture showed similar sensitivity values. In a study by Cohen et.al., AI was able to detect knee joint effusion, including lipo-hemarthrosis, with a sensitivity and specificity of 74.5% and 73%, respectively [10], showing higher sensitivity values compared to current findings with BoneView (42%) sensitivity and similar to those with RBfracture (75%). Similarly, Kim et. al. showed a sensitivity value of 82.0% [11], which is higher than that by Boneview and similar to that by RBfracture. In addition, Won et.al. found a sensitivity value of 82.0% [9]. In our study, RBfracture showed similar sensitivity values compared to previous studies [9], [10], [11]. On the contrary, BoneView showed higher specificity values compared to previous studies [9], [10], [11], whereas RBfracture showed similar [9], [11] or higher [10] values compared to previous studies, highlighting the potential of AI algorithms to rule out normal knee radiographs with no knee joint effusion. Comparing AI results to human observer values shows lower sensitivity for both AI algorithms, although the CIs overlap with the RBfracture values. On the contrary, both BoneView and RBfracture showed higher specificity than human observers. [16] In the presence or absence of injury-associated findings, BoneView maintained perfect specificity (1.00) in both classes, whereas RBfracture’s specificity dropped from 0.94 to 0.60 in injury cases, suggesting increased false positives. The ensemble analysis supports the interpretation that the moderate kappa primarily reflects the two algorithms operating at different points on the sensitivity-specificity trade-off: BoneView identified only one effusion that RBfracture missed. Combining the two algorithms therefore offers negligible diagnostic gain over RBfracture alone for effusion detection.

When the predictions from the AI algorithms for knee joint effusion and injury are combined, sensitivity is improved compared to our previous work which reported AI algorithm sensitivity values in injury detection. This comparison was conducted as an exploratory clinical workflow analysis. Higher sensitivity for both AI algorithms indicates improved detection of injury cases, likely including subtle injuries not yet apparent on plain radiographs that may be suggested by the presence of effusion or ligamentous injuries that may present with knee joint effusion but no obvious bony changes. This enhanced sensitivity has significant implications for patient outcomes in emergency radiology settings, where missing an injury could delay treatment, prolong recovery, or lead to complications such as joint instability or chronic pain. For instance, early detection of subtle injuries through AI-assisted identification of knee joint effusions could prompt timely advanced imaging (*e.g.*, MRI or CT), potentially improving functional outcomes and reducing the risk of long-term morbidity. In addition, a clinically more meaningful signal is the improvement in negative predictive values (NPVs) (0.98 for BoneView, 0.97 for RBfracture) compared to knee joint effusion detection alone (0.57 and 0.74, respectively), which suggests the combined model is most useful as a high-confidence rule-out tool. The negative combined prediction becomes a more reliable indicator that the patient has neither injury nor effusion findings, which are important in emergency triage. On the other hand, the trade-off of this combined approach is a notable decrease in specificity and positive predictive value. This increase in false positives could lead to overdiagnosis, resulting in unnecessary additional imaging with added cost and radiation exposure, patient anxiety, or resource strain in emergency departments. Increased workload from reviewing false-positive cases could overwhelm radiologists, potentially leading to diagnostic fatigue or delays in reporting true-positive cases. Thus, balancing these trade-offs is critical.

In this study, not all lateral knee projections were taken with a cross-table lateral projection, which may have hidden some cases with lipohemarthrosis. From all the lateral knee projections, as many as 28% were taken with a rolled lateral knee projection. The cross-table lateral projection should be used in traumatic knee projections because it results in less compression of the suprapatellar region due to less flexion compared to the rolled lateral projection [16]. In this study, the patient positioning did not have significant differences in diagnostic performance.

Some limitations of the current study warrant discussion. First, potential bias in the ground truth as it depends on the subjective judgment of two board-certified radiologists and, although arbitrated, reflects the moderate inter-reader agreement known for radiographic effusion detection. Second, we did not measure effusion volume, so we could not assess spectrum bias (i.e., whether algorithms preferentially detect larger effusions). Third, radiographs were used as the ground truth, so the results reflect the AI's ability to identify radiographically apparent disease rather than the true disease status, which CT or MRI would better capture. Fourth, this study uses a retrospective sample from a previously published study, which may introduce selection bias. Fifth, this study had a small sample size, impacting the robustness of the estimates. Finally, a key limitation in AI research in radiology is that, by the time of publication, advancements in AI software development often result in newer, improved versions being available.

In this study, two commercially available AI algorithms demonstrated different operating points for knee joint effusion detection from radiographs: BoneView functioned as a high-specificity classifier, while RBfracture achieved higher sensitivity. Combining injury and effusion predictions increased sensitivity for both algorithms, at the cost of specificity and positive predictive value. These exploratory findings should be interpreted in the context of the limited sample size and require prospective validation.

## CRediT authorship contribution statement

**Jarno T. Huhtanen:** Writing – review & editing, Writing – original draft, Visualization, Methodology, Investigation, Data curation, Conceptualization. **Mikko Nyman:** Writing – review & editing, Investigation, Data curation. **Roberto Blanco Sequeiros:** Writing – review & editing, Resources, Methodology, Conceptualization. **Seppo K. Koskinen:** Writing – review & editing, Methodology, Conceptualization. **Tomi K. Pudas:** Writing – review & editing, Methodology, Conceptualization. **Sami Kajander:** Writing – review & editing, Methodology, Conceptualization. **Pekka Niemi:** Writing – review & editing, Methodology, Conceptualization. **Hannu J. Aronen:** Resources, Methodology, Funding acquisition, Conceptualization. **Jussi Hirvonen:** Writing – review & editing, Writing – original draft, Visualization, Supervision, Project administration, Methodology, Investigation, Formal analysis, Data curation, Conceptualization.

## Study ethics

This retrospective study received ethical approval from the Ethics Committee of the University of Turku (ETMK Dnro: 38/1801/2020). The research complied with the Declaration of Helsinki, and due to its retrospective nature, the need for informed consent was waived by the Ethics Committee of the Hospital District of Southwest Finland.

## Declaration of Generative AI and AI-assisted technologies in the writing process

In preparing this manuscript, the authors employed Claude Opus 4.7 and Grammarly Pro to improve the grammatical accuracy and semantic clarity of some sections of the text. The authors then reviewed and revised the content as needed, taking full responsibility for the final published version.

## Funding

This research was supported by the 10.13039/501100008490Radiological Society of Finland.

## Declaration of Competing Interest

The authors have no conflicts of interest to declare that are relevant to the content of this article.

## Data Availability

Data cannot be publicly shared because of the national legislature on the privacy of patient data.

## Footnotes

Appendix A Supplementary data associated with this article can be found in the online version at doi:10.1016/j.ejro.2026.100760.
