# Supplementary material for: AI detection of knee joint effusion from radiographs: Comparative accuracy of two commercial algorithms
Source: Eur J Radiol Open. 2026 May 8;16:100760. doi: 10.1016/j.ejro.2026.100760 (PMC13186055; doi:10.1016/j.ejro.2026.100760)
Supplement: Supplementary file 1 — Supplementary material [file mmc1.docx]

Supplementary Table 1. AI Performance in Different Lateral Projections

|  |  | TP | TN | FP | FN | Sensitivity | Specificity | Cohen’s Kappa |
| --- | --- | --- | --- | --- | --- | --- | --- | --- |
| BoneView (95% CI) | Cross-table lateral | 20 | 44 | 0 | 31 | 0.39 (0.27–0.53) | 1.00 (0.92–1.00) | 0.46 |
|  | Rolled lateral | 9 | 10 | 0 | 9 | 0.50 (0.29–0.71) | 1.00 (0.72–1.00) | 0.58 |
| RBfracture (95% CI) | Cross-table lateral | 39 | 41 | 3 | 12 | 0.77 (0.63–0.86) | 0.93 (0.82–0.98) | - |
|  | Rolled lateral | 13 | 8 | 2 | 5 | 0.72 (0.49–0.88) | 0.80 (0.49–0.94) | - |
